# Rickets manifestations in a child with metaphyseal anadysplasia, report of a spontaneously resolving case

**DOI:** 10.1186/s12887-021-02716-x

**Published:** 2021-05-22

**Authors:** Patricia Diaz Escagedo, Melissa Fiscaletti, Patricia Olivier, Chloé Hudon, Valancy Miranda, Marie-Claude Miron, Philippe M. Campeau, Nathalie Alos

**Affiliations:** 1 Bone and mineral Clinic, Sainte-Justine Hospital Center, Department of Pediatrics, 3175 Chemin de la Côte-Sainte-Catherine, Montreal, QC Canada; 2 Medical Genetics Service, Sainte-Justine Hospital Center, Department of Pediatrics, 3175 Chemin de la Côte-Sainte-Catherine, Montreal, QC Canada; 3 Radiology Department, Sainte-Justine Hospital Center, Department of Pediatrics, 3175 Chemin de la Côte-Sainte-Catherine, Montreal, QC Canada; 4 Research Center, Sainte-Justine Hospital Center, 3175 Chemin de la Côte-Sainte-Catherine, Montreal, QC Canada

**Keywords:** Metaphyseal anadysplasia, Regressive course, Rickets mimicking presentation disease, Autosomal dominant variant

## Abstract

**Introduction:**

Rickets is not an unusual diagnosis for pediatricians even currently in developed countries. Children typically present with leg bowing, enlargement of wrists, rachitic rosary (swelling of costochondral junctions) and/or waddling gait. But not every child with growth delay and enlarged metaphyses is diagnosed with rickets. Metaphyseal anadysplasia (MAD) is a disorder of variable severity with metaphyseal flaring and irregularities, without vertebral abnormalities. MAD is characterized by an early onset and a regressive course in late childhood without treatment, despite persistent short stature. Autosomal dominant or recessive variants in the matrix metalloproteinase 13 gene (*MMP13*) are responsible for these transient metaphyseal changes.

**Case presentation:**

We report a new pathogenic heterozygous variant in *MMP13 *(NM_002427.4: c.216G>C, p.Gln72His*)* in a toddler, initially thought to have rickets, and his father, with MAD phenotypes. Additionally, we review the seven reported *MMP13* variants.

**Conclusion:**

One should keep a wide differential diagnosis in cases of suspected rickets, including skeletal dysplasias which might have a regressive course.

**Supplementary Information:**

The online version contains supplementary material available at 10.1186/s12887-021-02716-x.

## Introduction

Rickets is a condition that general pediatricians should be familiar with as its prevalence has increased in the past 20 years [1, 2]. It is characterized by leg bowing, enlargement of wrists, rickety rosary (enlarged costochondral junctions) and poor growth. Waddling gait, motor delay and bone pain can also be observed. Radiological findings observe concave “moth-eaten” appearance of the metaphyses especially at the radius, ulna, distal femur and proximal tibia. In the chest X-ray, costochondral enlargement is seen. Different etiologies exist, the most common being nutritional rickets, due to Vitamin D deficiency. Metaphyseal dysplasia, however, can present similar clinical manifestations to nutritional rickets and should be retained as a differential diagnosis.

Metaphyseal anadysplasia (MAD) was first described in 1971 by Wiedemann and Spranger in a patient with regressive course of metaphyseal dysplasia [3]. In 1991, Maroteaux described four cases of metaphyseal anadysplasia (ana-, is a greek prefix meaning return) [4] and in 1992, the disease name was widely accepted in the International classification of osteochondrodysplasias [5]. It is a bone disorder with radiological and clinical skeletal changes categorized in two types. These varieties of MAD have been defined according to gene inheritance and transmission, as well as phenotype severity. MAD type 1(MIM #600108) is caused by an autosomal dominant variant in exon 2 of *MMP13*. Individuals with MAD type 1 can present with short adult stature. In contrast, MAD type 2 (MIM #613073), caused by autosomal recessive variants in *MMP13* or *MMP9*, has a milder phenotype with individuals achieving normal adult stature. Differential diagnoses at the initial presentation include nutritional and hypophosphatemic rickets but once excluded, other metaphyseal chondrodysplasias with a worse course could be taken into consideration such as cartilage hair hypoplasia (MIM #250250), Jansen (MIM # 156400) and Schmid (MIM #156500) metaphyseal chondrodysplasias, or Spahr-type metaphyseal dysplasia (MIM#250400). The latter is also caused by an autosomal recessive variant in exon 5 of *MMP13* [6–9].

Heterozygous *MMP13* pathogenic variants are implicated in MAD type 1 which presents early in life with short stature, rhizomelic micromelia, varus deformity of lower extremities and severe long bone metaphyseal changes without vertebral or skull anomalies [9]. Bone of the lower limbs are more commonly affected than upper limbs; specifically, the knee and femoral neck. The prognosis is favourable with the dysplasia taking on a regressive course. The phenotype occurs and is restricted to periods of rapid growth. Some authors have described normalized radiological images at 3 years of age [10], others at 5 years of age [11]. Final adult height may be below the expected genetic potential [11, 12]. Here we present an individual and his father with MAD type 1 and a novel missense variant in *MMP13*.

## Case presentation

**Fig. 1:**
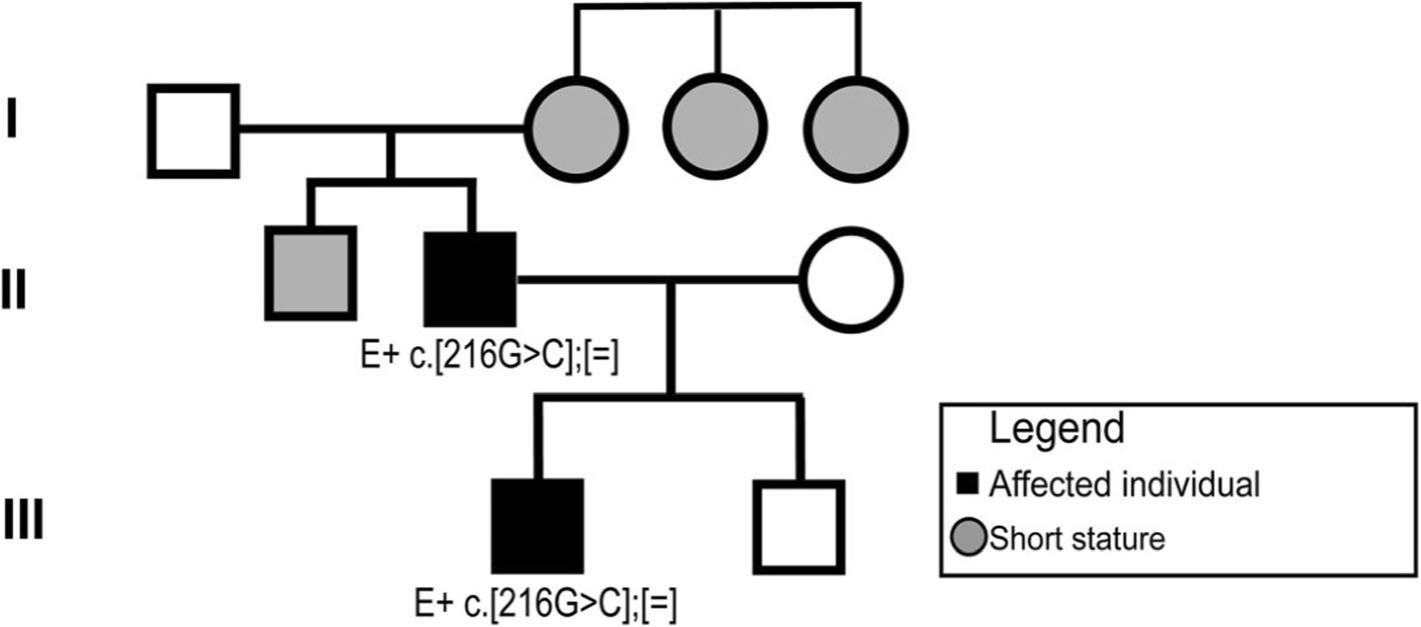
Familial pedigree. Our proband (III-1) and father (II-2) have the described variant c.216G > C;p.Gln72His. Proband’s uncle (II-1), and two of the paternal grandmother’s sisters (I-3 & I-4) may be also affected because of short adult stature but were not evaluated clinically or molecularly

**Fig. 2:**
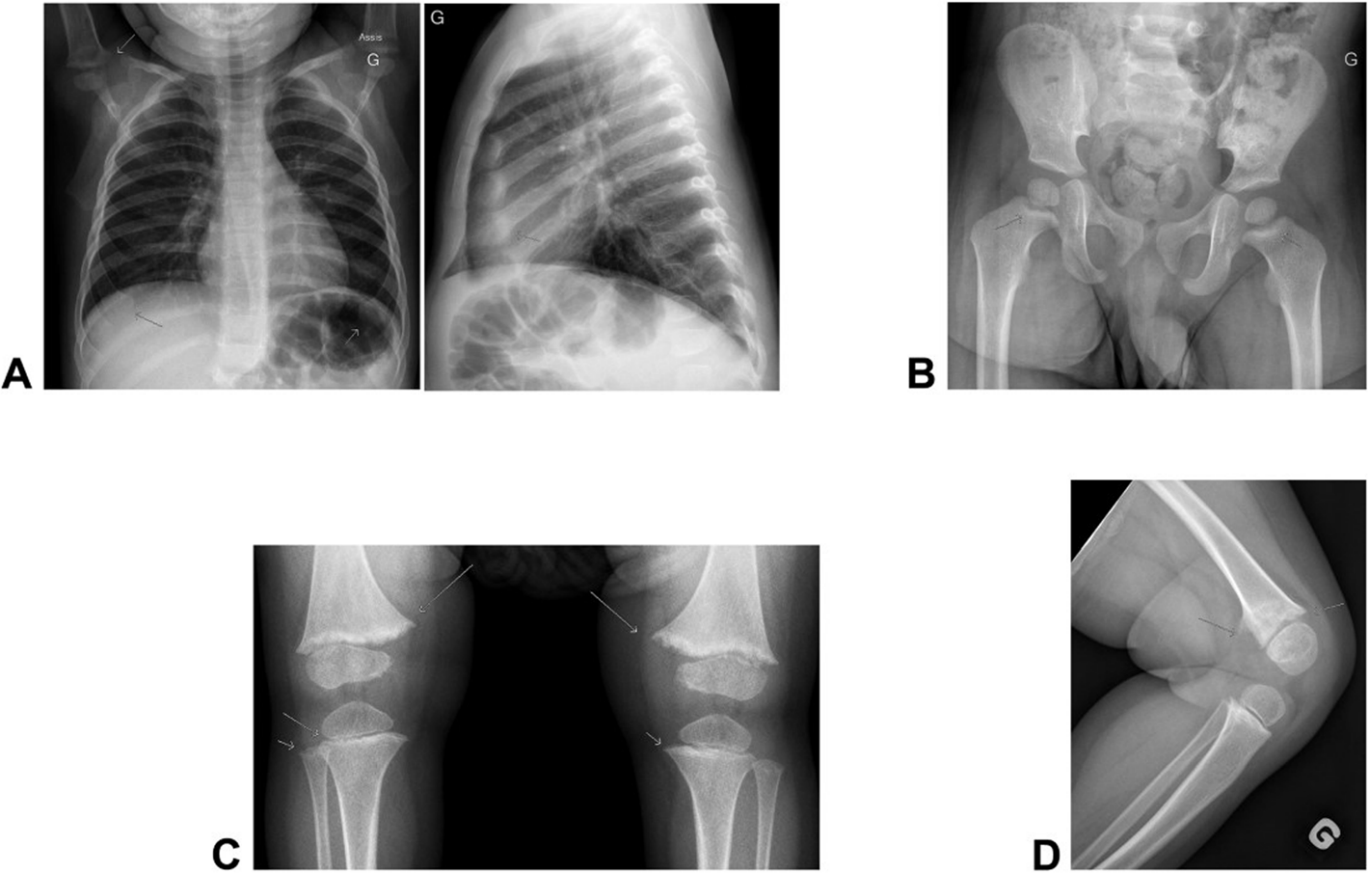
** a** Proband’s chest X-ray, at 18 months-old, revealing widening and irregularities of humeral metaphyses along with cupping and enlargement of anterior costochondral junctions. **b** Proband’s hip X-ray at 24 months with cupiliform aspect of femoral neck. **c** & **d** Proband’s knee X-ray at 18 months-old revealing fine-tooth-comb irregularities and widening of metaphyses

An 18-month-old boy (individual III-1 in Fig. 1) was referred to the Sainte-Justine University Hospital Center’s Bone Health Clinic for suspicion of rickets. A chest X-ray, initially done to rule out pneumonia, revealed incidental findings of humeral metaphyseal widening and cupping of anterior costochondral junctions suggestive of rickets (Fig. 2 a).

The toddler was the first-born child at term to a Cuban father and Canadian mother with weight and height at the 50th percentile. Psychomotor development was appropriate for age and independent walking was reached at 14 months. The mother reported that the child presented an occasional waddling gait and frequent falls. He had recurrent asthma-like episodes but no signs of bone pain or fractures. He had a normal diet for his age and was supplemented with daily 400 IU of cholecalciferol. His height curve followed the 1^srt^ centile.

While the maternal past-medical history was non-contributory, the proband’s paternal family history was notable for short stature. His father (individual II-2) was born with occult spina bifida and was followed by a pediatric endocrinologist for short stature during his childhood in Cuba. The father’s adult height is 165 cm (4th percentile, -1.7 SD). We were unable to obtain the father’s Cuban medical chart or pediatric X-ray images. Extended paternal family history revealed short stature in multiple family members including two sisters of the paternal grandmother who are described as having a height of less than 150 cm (individuals I-3 and I-4). Clinical assessment of extended family members was not feasible as they currently reside in Cuba. Our proband has a 10-month-old brother (individual III-2) that has been recently evaluated. He did not show any metaphyseal changes neither clinically or radiologically and his growth was at the 15th percentile. Moreover, his genetic testing was negative for the *MMP13* missense variant found in his brother and father.

Physical examination revealed typical signs of rickets with bilateral genu varum, rachitic rosary, enlarged wrist metaphyses and non-ossified anterior fontanelle. Height for age was at the 0.4th percentile (-2.6 SD), below his familial target (172 cm, 25th percentile) and height velocity was decreased (10 cm/year - normal height velocity for this group age is 20 cm/year).

An irregular and cupiliform aspect of the femoral neck was reported on pelvis X-ray (Fig. 2b). On knee X-ray, wide and irregular metaphyses resembling a fine-tooth comb were also described in distal femoral, proximal tibial and proximal right fibular metaphyses (Fig. 2 c & d) yet bone mineral density (by Lunar prodigy, GE) was in the normal range for age and gender. Initial laboratory findings revealed eucalcemia, euphosphatemia with suppressed parathyroid hormone (PTH), normal urine calcium on creatinine ratio and TmP/GFR (Table 1). He had normal renal and liver function tests and celiac screening was negative. Vitamin D status was sufficient with 25(OH)vitamin D level at 82 nmol/L. Serum levels of 1,25 (OH) vitamin D, vitamin B6 and C-terminal FGF23 were all in the normal range (Table 1). The initial serum alkaline phosphatase (ALP) activity level was low, suggesting hypophosphatasia as a possible differential diagnosis.

**Table 1:** Laboratory results, initial and follow up

	2018/08/10	2018/08/15	2018/09/13	2018/12/20	2019/03/14
**Total calcium (2.29–2.63) mmol/L**	2.27	2.39	*N/A*	2.41	2.5
**Ionised calcium (1.22–1.37) mmol/L**	*N/A*	1.22	*N/A*	1.21	1.27
**Phosphatemia (1.38–2.19) mmol/L**	1.59	1.7	1.81	1.93	1.77
**Magnesium (0.65–1.1) mmol/L**	0.81	*N/A*	0.8	0.93	0.8
**Alkaline phosphatase (156–369) U/L**	**152 (↓)**	**146 (↓)**	175	202	221
**PTH (1.3–6.8) pmol/l**	< 0.3	*N/A*	*N/A*	1.4	0.8
**25(OH)Vitamin D (> 75) nmol/L**	88	*N/A*	*N/A*	85	*N/A*
**1.25(OH)Vitamin D (63–228) pmol/L**	*N/A*	442	*N/A*	*N/A*	*N/A*
**Ca/creat (0–1) mmol/mmol**	0.33	*N/A*	0.32	*N/A*	0.56
**TRP %**	***N/A***	*N/A*	90	*N/A*	87
**Vitamine B6**	*N/A*	*N/A*	39	*N/A*	*N/A*
**FGF23 (19–114) U/mL**	*N/A*	*N/A*	*N/A*	*N/A*	75
**TSH (0.6–4.4) mU/L**	*N/A*	1.9	*N/A*	*N/A*	2.64
**IGF-1 (29–189) ng/mL**	*N/A*	*N/A*	*N/A*	*N/A*	121^a^

^a^ z-score 0.88

A genetic rickets panel at Prevention Genetics covering fully the coding and splicing regions of 7 genes, and including the *ALPL* gene was requested and revealed a heterozygous variant of unknown significance (VUS) in *DMP1*, c.1466G > A; p.Arg489Gln. We determined that the heterozygous *DMP1* VUS in our patient was not causal of metaphyseal anomalies for 2 reasons: (1) Only homozygous loss of function variants in *DMP1* cause severe hypophosphatemic rickets with autosomal recessive inheritance [13–15], and (2) our patient’s phenotype did not correspond to the *DMP1* hypophosphatemic rickets phenotype previously described.

Additionally, the radiological diagnosis of rickets was unlikely since physes were not enlarged and metaphyseal band’s density was normal. Knowing that the genetic rickets panel was non-contributory, and that skeletal dysplasia can present as metaphyseal anomalies, a spondyloepimetaphyseal dysplasia genetic panel at CTGT was ordered (covering fully the coding and splicing regions of 54 genes). Noted variants included a single heterozygous variant in *HSPG2* (Exon 70 c.9326delA/His3109Profs*16) and a single heterozygous variant in *RNU4ATAC* (transcribed region n.48G > A). These genes are associated with Schwartz-Jampel syndrome and Roifman syndrome respectively, which are autosomal recessive disorders (i.e. both alleles require pathogenic variants) with very different manifestations from those presented by the proband. However, the panel revealed a heterozygous missense VUS in exon 2 of *MMP13*, c.216G > C/p.Gln72His in the proband. Segregation studies revealed the same variant in the father’s sample (individual II-2) confirming the autosomal dominant inheritance. The variant is predicted to be deleterious based on the following: (1) Amino acid (AA) 72 is highly conserved between species (2). This AA borders other AAs affected by known pathogenic variants in MMP13 leading to MAD (3). Multiple pathogenic prediction scores as DANN: 0.996, CADD: 24.4 and SIFT4G: 0 [16, 17] considered the variant as damaging (Table S1) [4] The variant is not present in gnomAD (v2.1.1).

## Discussions and conclusions

**Fig. 3:**
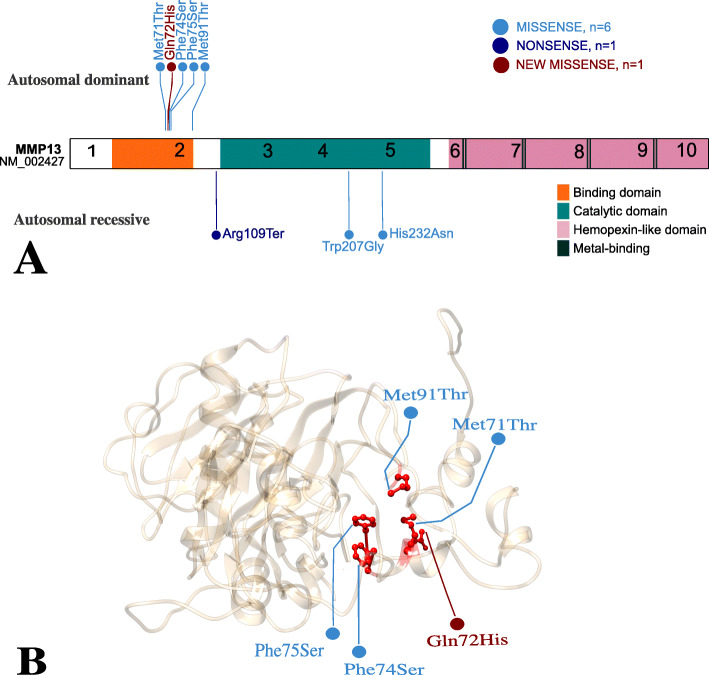
MMP13 protein structure and its 3 different domains: binding, catalytic and hemopexin-like. **a** The numbers correspond to the different exons. Known pathogenic variants are drawn in their location in the protein, according to inheritance pattern. There are 7 missense variants, including the one described in this paper and 1 nonsense variant. **b** A schematic diagram of MMP13 3D protein structure, the 5 pathogenic variants leading to MAD type 1 are between aa 71 and 75, in the binding domain. Model done with The Phyre2 web portal [20], representation done with Chimera [21].

We describe an individual and his father with MAD type 1 and a novel missense variant in *MMP13*. To our knowledge, there are only seven *MMP13* variants described in the literature. Six of them lead to MAD type 1 or 2. Autosomal dominant variants in exon 2 cause MAD type 1 and present a relatively severe course with reduced adult stature. The variant described by Kennedy et al. resulted in spondyloepimetaphyseal dysplasia, Missouri type (SEMDMO) (MIM #60211) which is also dominantly inherited and is classified as MAD type 1 [18, 19]. Autosomal recessive variants involved in MAD type 2 present a milder course and a normal height in adulthood [9, 10, 12]. Three patients have been published with metaphyseal dysplasia, Spahr type caused by the same autosomal recessive variant in exon 5 but leading to a chronic disease [6, 7]. (Fig. 3/ Table 2).

**Table 2:** Previously reported *MMP13* pathogenic variants and the new variant we report here: inheritance, exon, amino acid change and clinical features

Description	Gene	Inheritance	Exon	Mutation	Amino acid change	Disease	Bowed leg/ Wadling gait	Stature range (SD)	Articular pain
Diaz Escagedo et al. (this report)	*MMP13*	AD	2	missense	p.Gln72His	MAD 1	Yes/Yes	-1.7 to -2.6	No
Song et al., 2019	*MMP13*	AD	2	missense	p.Met71Thr	MAD 1	ND/Yes	No	No
Lausch et al., 2009	*MMP13*	AD	2	missense	p.Phe74Ser	MAD 1	Severe/ND	-1.72 to -2.58	No
Lausch et al., 2009	*MMP13*	AD	2	missense	p.Met91Thr	MAD 1	Yes/ND	+ 1.6 to -2.89	No
Kennedy et al., 2005	*MMP13*	AD	2	missense	p.Phe75Ser	MAD 1^a^	Yes/ND	ND	No
Li et al., 2015	*MMP13*	**AR**	2	**nonsense**	p.Arg109Ter	MAD 2	Yes/Yes	-1.9 to -2.6	No
Lausch et al., 2009	*MMP13*	**AR**	4	missense	p.His232Asn	MAD 2	Yes/Yes	-0.23 to -1	Yes
Bonafé et al., 2014/Tadros et al., 2017	*MMP13*	**AR**	5	missense	p.Trp207Gly	MDST	Yes/ND	-1.31 to -3.85	Yes

*AD *Autosomal Dominant, *AR *Autosomal Recessive, *MAD 1 *Metaphyseal Anadysplasia type 1, *MAD 2*  Metaphyseal Anadysplasia type 2, *MDST *Metaphyseal dysplasia, Spahr type, *ND *not described

^a^as describe in the text, MAD 1 and SEMD MO = spondyloepimetaphyseal dysplasia, Missouri type are classified as the same disease. Bonafe, Cormier-Daire [18]

*MMP13* encodes matrix metalloproteinase 13 (MMP13) expressed in hypertrophic chondrocytes and osteoblasts of growing bones during periods of rapid growth (e.g. embryogenesis, infancy). MMP13 protein is a zinc dependent proteolytic enzyme that works synergistically with matrix metalloproteinase 9 (MMP9) as major proteoglycanase and collagenase in hypertrophic chondrocytes. Degradation of both collagen type II (most frequent chondral collagen) and proteoglycan occur only in the very last stages of chondrocytes differentiation just prior to vascular invasion. Matrix metalloproteinases cleave fibrillar collagen between aa 775 and 776 within their triple helical regions, these fragments will then be denatured and degraded by gelatinases like MMP9 [22–24]. Lausch et al. reported that variants in the propeptide or binding domain (Fig. 3) will cause the disruption of MMP13’s tertiary structure and will result in its premature activation. Activated MMP13 degrades both itself (autocatalytic self-inactivation) and MMP9. When chondrocytes enter the hypertrophic stage, they expand 5–10 fold, secrete collagen type X and deposit vascular endothelial growth factor (VEGF) into the matrix as well as produce ALP and calcify their extra-cellular matrix (ECM) [25]. Additionally, they express MMP13 prior to apoptosis, leaving an empty lacuna surrounded by a calcified matrix that is subsequently remodeled into bone [23]. Loss of MMPs prolongs the chondrocytes’ hypertrophic stage. Hypertrophic chondrocytes are physiological, but their delayed apoptosis results in a disorganised ECM with unaligned chondrocytes leading to enlarged growth plates. These changes are seen clinically and radiologically and could be mistaken for rickets. As these changes regress over time, studies in mice have suggested that other MMPs might compensate for the lack of Mmp13 or Mmp9 [22, 24]. Osteoblast differentiation and function depend on the correct remodeling of cartilage. In the endochondral bones of Mmp13^−/−^ mice, altered residual cartilage matrix causes impaired osteoblast differentiation and subsequent improper bone deposition [23]. This phenomenon may explain the low serum ALP activity initially seen in our patient.

To conclude, this case supports the importance of considering skeletal dysplasia in the differential diagnosis of children who present with atypical rickets manifestations, as already highlighted by Michalus et al. [26]. Furthermore, this case underscores the role of radiological follow-up, and, in selected cases, molecular testing for skeletal dysplasia in children who present rachitic-like features with an atypical clinical course.

We report a novel pathological variant in *MMP13* associated with MAD type 1 in a young boy and his father who presented with short stature and a skeletal phenotype mimicking rickets. The autosomal dominant inheritance of the *MMP13* missense variant in exon 2 is consistent with our proband’s family history, and his clinical and radiological presentation. Metaphyseal dysplasia remains an important differential diagnosis in children presenting metaphyseal changes classically described in rickets.

## Supplementary Information

Additional file 1: **Table S1. **Pathogenicity predictions scores for c.216G>C/p.Gln72His on MMP13.

## Data Availability

Data sharing is not applicable to this article as no datasets were generated or analysed during the current study.

## Abbreviations

MAD: Metaphyseal anadysplasia
*MMP13*: Matrix metalloproteinase 13 gene
*MMP9*: Matrix metalloproteinase 9 gene
PTH: Parathyroid hormone
ALP: Alkaline phosphatase
VUS: Variant of unknown significance
DMP1: Dentin Matrix Acidic Phosphoprotein 1 gene
MMP13: Matrix metalloproteinase 13 in mice
MMP9: Matrix metalloproteinase 9 in mice
MMP13: Matrix metalloproteinase 13 knockout mice
SEMDMO: Spondyloepimetaphyseal dysplasia , Missouri type
MMP13: Matrix metalloproteinase 13
MMP9: Matrix metalloproteinase 9
AA: Amino acid
