# Supplementary material for: Rickets manifestations in a child with metaphyseal anadysplasia, report of a spontaneously resolving case
Source: BMC Pediatr. 2021 May 22;21:248. doi: 10.1186/s12887-021-02716-x (PMC8140414; doi:10.1186/s12887-021-02716-x)
Supplement: Supplementary file 1 — Table S1. Pathogenicity predictions scores for c.216G>C/p.Gln72His on MMP13. [file 12887_2021_2716_MOESM1_ESM.docx]

**Table S1:** Pathogenicity predictions scores for c.216G>C/p.Gln72His on MMP13

|  |  | | **MMP13: c.216G>C / p.Gln72his** | | |
| --- | --- | --- | --- | --- | --- |
| **Algorithm** | |  | **Score** |  | **Prediction** |
| DANN^1^ | |  | 0.996 |  | Damaging |
| SIFT4G^2^ | |  | 0 |  | Intolerant |
| CADD^2^ | |  | 24.4 |  | Damaging |

^1^ Quang D, Chen Y, Xie X. DANN: a deep learning approach for annotating the pathogenicity of genetic variants. Bioinformatics. 2015;31(5):761-3

^2^ Richards S, Aziz N, Bale S, Bick D, Das S, Gastier-Foster J, et al. Standards and guidelines for the interpretation of sequence variants: a joint consensus recommendation of the American College of Medical Genetics and Genomics and the Association for Molecular Pathology. Genet Med. 2015;17(5):405-24
